# Outbreaks of iatrogenic botulism in Europe: Combating off-label medical use of Botulinum Neurotoxin (BoNT) in bariatric procedures

**DOI:** 10.1016/j.nmni.2023.101152

**Published:** 2023-05-22

**Authors:** Nityanand Jain, Edouard Lansiaux, Umut Yucel, Swantje Huenermund, Stella Goeschl, Patricia Schlagenhauf

**Affiliations:** Faculty of Medicine, Riga Stradinš University, 16 Dzirciema Street, Riga, LV-1007, Latvia; Lille University School of Medicine, 2 Avenue Eugène Avinée, 59120, Loos, Lille, France; Faculty of Medicine, Bahcesehir University, Istanbul, 34349, Turkey; Faculty of Medicine, Riga Stradinš University, 16 Dzirciema Street, Riga, LV-1007, Latvia; Department of Cardiology and Intensive Care Medicine, Helios Clinic Erfurt, Erfurt, 99089, Germany; Medical University of Vienna, Vienna, 1090, Austria; University of Zürich, Institute for Epidemiology, Biostatistics and Prevention, WHO Collaborating Centre for Travellers' Health, Department of Global and Public Health, MilMedBiol Competence Centre, Zürich, Switzerland

**Keywords:** Botulism, Europe, Iatrogenic, Neurotoxin, Weight loss

In late March 2023, the World Health Organization (WHO) issued a disease outbreak notification regarding an iatrogenic outbreak of botulism in 87 patients who underwent bariatric (weight-loss) procedures at two Turkish hospitals in Istanbul and Izmir [1,2]. However, authorities believe there may be more victims to come, with those affected estimating that more than 250 may have been affected by the current outbreak. The patients reportedly underwent the procedures between February 3 and March 1, 2023. Since then, the authorities have suspended activities at these centres and launched an internal investigation. It has been determined that all affected patients received intragastric botulinum neurotoxin (BoNT), an off-label use for a well-known product in cosmetic dermatology [2].

The European Centres for Disease Prevention and Control (ECDC) has noted that the iatrogenic botulism cases linked to the current outbreak have been reported in Germany, Austria, France, Switzerland, and Turkiye [2]. All affected patients are reported to be adults with the majority being middle-aged women. Multiple patients have been admitted to the intensive care unit due to worsening condition. The reported symptoms of these patients have been summarized in Fig. 1.

**Fig. 1:**
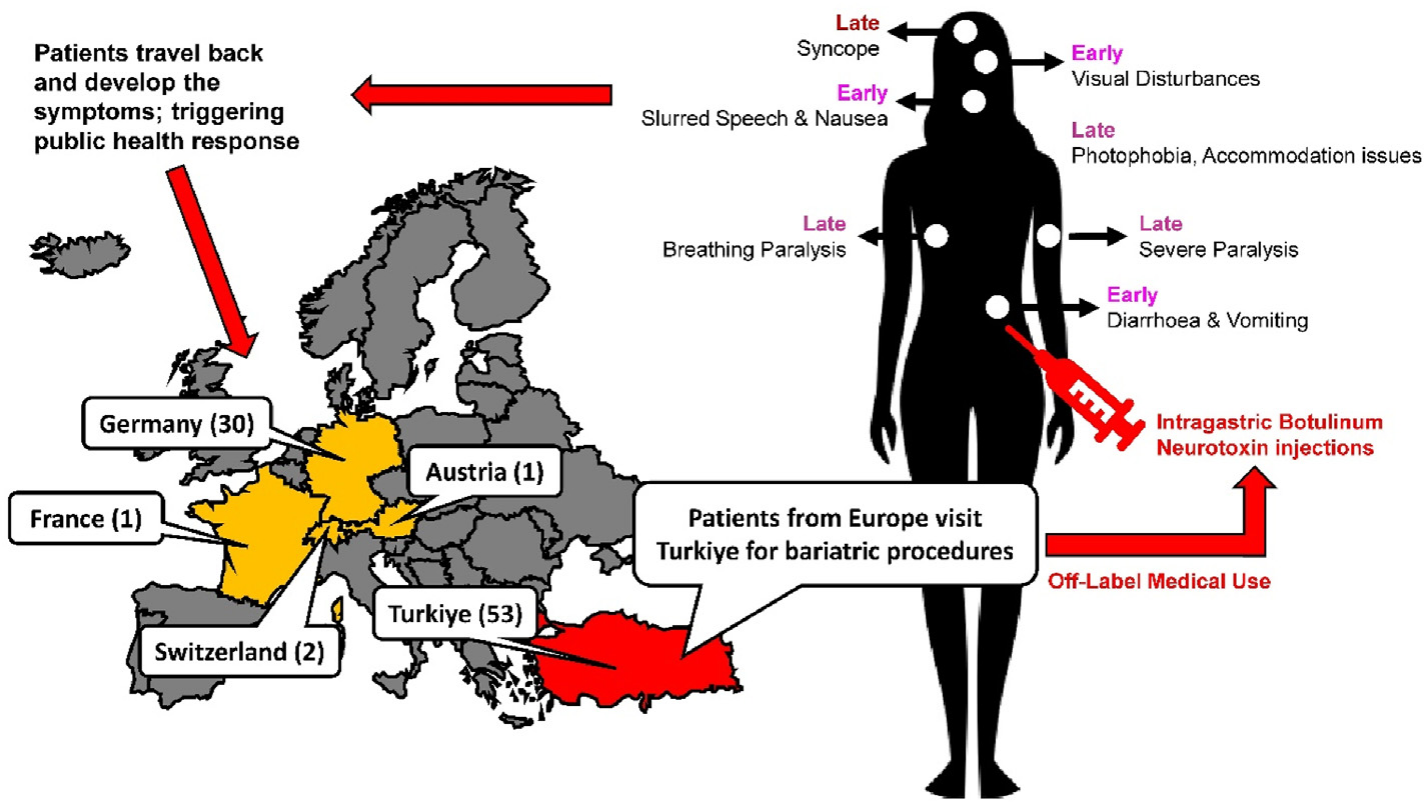
A schematic demonstrating the chain of events during the current outbreak. Patients from multiple European countries visited Turkiye for intragastric botulinum neurotoxin injections (for weight loss). The patients travel back to their home countries where they developed the depicted early (within first three days) and late (fourth day onwards) complications. This triggered a public health response in the European countries including Germany, France, Switzerland, and Austria. Brackets depict the number of confirmed cases in each country. Note – the authors remain neutral in regards with territorial disputes. The images were adapted from the Noun Project.

## 1 What is botulinum Neurotoxin?

As one of the most poisonous toxins, Botulinum neurotoxins are metallo-endoproteases produced by gram positive, anaerobic *Clostridium* spp. Due to the toxin's ability to inhibit the release of neurotransmitter (acetylcholine) at the striated neuro-muscular junction, it is widely used for disorders characterized by muscular dystonia or hypertonia [3]. However, its use in cosmetology for aesthetic purposes is perhaps the best known with nearly three million injections used annually [4]. The toxin is cleaved into a two-chain molecule (light and heavy chain) connected by a disulphide bridge. The heavy chain binds to the presynaptic receptors and is responsible for translocation of the light chain into the neuronal cytoplasm. The light chain possesses the proteolytic activity and act as a zinc endopeptidase [5].

There are seven known serotypes (A to G) with more than 40 subtypes described based on variations in amino acid sequences [6]. Recently, an eighth serotype (X) has been described [7]. Serotypes C and D are associated with botulism in animals and birds while serotypes A, B, E, and F (rarely) are linked to food-borne botulism in humans [8]. Among these serotypes, the most potent is Botulinum toxin A (BTA) which is also used for medical purposes. In Turkiye, there are three BTA products that are approved (Table 1).

**Table 1:** List of Botulism Toxin products licensed by the Turkish Medicines and Medical Device Agency.

Product^a^	BOTOX®	Dysport®	NABOTA®
**Manufacturer**	Allergan Pharmaceuticals	Gen Pharmaceuticals and Health Products	Seltek Ilac Marketing and Foreign Trade
**Active Ingredient**	Onabotulinumtoxin A	Clostridium botulinum Type A hemagglutinin complex	Botulism Toxin type A
**Excipients**	• Human Albumin	• Human Albumin	• Human Albumin
	• Sodium Chloride	• Lactose (cattle)	• Sodium Chloride
**Units per vial**	50/100/200 units	500 units	100 units
**Preparation**	Lyophilized Powder for Solution for Injection	Powder for solution for injection	Lyophilized powder for solution for injection
**Usage Form**	By injections intramuscularly, into the wall of the urinary bladder or into the skin	By injections intramuscularly, intradermally, or subcutaneously	By injections subcutaneously
**Storage**	Refrigerator (2 ​°C–8 ​°C) or freezer (−5 ​°C or below)	Refrigerator (2 ​°C–8 ​°C). Do not freeze.	Refrigerator (2 ​°C–8 ​°C). Do not freeze.
**Indications**	• Paediatric cerebral palsy	• Blepharospasm	• Frowning
	• Blepharospasm	• Hemifacial spasm	• Correction of facial lines
	• Cervical dystonia	• Spasmodic torticollis	
	• Focal spasticity after stroke	• Axillary hyperhidrosis	
	• Chronic migraine	• Focal spasticity after stroke	
	• Correction of facial lines	• Paediatric cerebral palsy	
		• Correction of facial lines	
**Absolute Contraindications**	• Allergic to components	• Allergic to components	• Allergic to components
	• Injection site infection		• Injection site infection
	• Urinary tract infections		• Neuromuscular junction disorders
			• Cervical dystonia
			• Urinary tract infections
			• Shouldn't be used in patients younger than 20 years old
**Pregnancy/Breastfeeding**	Contraindicated	Contraindicated	Must use birth control methods. Contraindicated for breastfeeding.
**Very Common Side Effects (1 in 10 patients)**	• Viral infections	• Droopy upper eyelid	• Droopy upper eyelid
	• Ear infections	• Muscle weakness	• Dizziness
	• Urinary tract infections	• Difficulty swallowing	
	• Drooping eyelids	• Dry mouth	
	• Pain	• Injection site reactions	
	• Muscle Weakness	• Headache	
	• Difficulty swallowing		
	• Urinary retention		
**ATC Code**	M03AX01	M03AX01	M03AX01
**Licensed since**	December 2022 (50 units)	July 2002	April 2021
	October 2010 (100 units)		
	December 2022 (200 units)		
**Barcode**	8699490550618 (50 units)	8699783790110	8680869372016
	8699490579015 (100 units)		
	8699490550625 (200 units)		
**Product Catalogue (in Turkish)**	http://www.ilacabak.com/pdf/9d3dfeea956936a.pdf	http://www.ilacabak.com/pdf/be7ade73e982b69.pdf	http://www.ilacabak.com/pdf/2b832667775c866.pdf

a Data source - List of Licensed Medicinal Products for Human Use 28^th^ April 2023 (available from https://www.titck.gov.tr/dinamikmodul/85; accessed 05th May 2023).

Apart from food-borne botulism (caused due to improper processing, storage, and transportation), there are other forms of botulism including infant botulism, wound-borne botulism, and the less common iatrogenic botulism. Iatrogenic botulism typically occurs due to accidental overdose or use of contaminated (sub-standard) products [8]. In the current outbreak, the Turkish authorities have suspected both the use of high dose injections that led to incessant vomiting causing subsequent electrolyte disbalances, and the use of illegal generics with non-standardized dosages and recommendations guidelines as the root cause of the crisis.

The effects of the toxin usually occur 24–72 hours post injection, peaking within the first two weeks, followed by gradual diminishing of its effects over the next three to four months [9]. Based on extrapolations from mouse models, it has been suggested that a dose of 33 units/Kg can cause systemic toxicity while a dose of 38–42 units/Kg is lethal for humans [10]. Identification of patients suffering from botulism is based on the clinical presentation and documented medical history of use of unlicensed injections or undergoing off-label procedures [11]. Laboratory and radiologic investigations may not be warranted in all cases. Respiratory failure is the primary cause of death and patients should be monitored closely [11]. Management of the affected patients require administration of botulinum antitoxin in suspected and confirmed cases of iatrogenic botulism as soon as possible.

## 2 Are botulinum injections effective in weight loss?

The literature is sparse on the clear benefits of intragastric botulinum injections and weight loss. From our literature search, we found that 13% of the total output on this topic comes from Turkish researchers and clinicians.^2^ In a recent prospective study from Turkiye, 56 obese patients received 8–10 1ml injections of BTA (25 units per injection) into the gastric antrum during upper gastrointestinal endoscopy after 6–12 hours of fasting [12]. Within the first two to three months after the procedure, the authors noted a mean weight loss of 9 kg. It is pertinent to note that the patients were advised to follow a specific diet after the procedure. Interestingly, most patients felt a decrease in appetite and early satiety, with half of the patients being satisfied with the results [12]. There were no reports of serious side effects in the study.

Another retrospective study reported significant reduction in weight loss when injecting BTA in combination with liraglutide (a glucagon-like peptide-1 agonist used to treat diabetes and chronic obesity) than BTA alone [13]. Multiple other studies have reported on the benefits of combining BTA intragastric injections with low-calorie diet [[14], [15], [16]]. In a study from Jordon, the authors noted that although BTA injections are associated with lesser weight loss in comparison with gastric balloons, shorter procedure duration and fewer postoperative complications may make BTA injections a favourable treatment modality [17].

However, the results from these studies need cautious interpretations. Firstly, the studies have variations in the site of injections (antrum vs fundus vs body of the stomach), dosage of injections, diet control methods, follow-up time points, and small number of participants included in the study (mostly <100 patients). A recent meta-analysis of randomized controlled trials reported that the procedure might be successful only if ​≥ ​200 units of BTA are administered at multiple sites in the gastric wall along with diet control [18]. Yet again, the authors cautioned interpretation due to low sample size, need for subgroup analysis (leading to higher chances of Type I errors), and limited power of the included studies. Such an effect is clearly seen when one considers pooled data from studies whereby there is no mean difference for absolute weight loss and BMI reduction when comparing BTA injections with saline injections and/or control subjects [18,19]. Furthermore, long term follow-ups (>6 months) are scarcely reported that limit our understanding of the possible side effects [20].

Apart from its obvious benefits of shorter durations, less costly, fewer complications, a study reported that patients post-BTA injections felt less anxiousness, positive personality, less hostility than before the procedure [21]. However, the patients reported higher levels of depression post-injections, which was found to be statistically non-significant. According to the Food and Drug Authority (U.S. FDA), such intragastric use of BTA would classify the procedure as Level 1 risk which requires the procedure to result in at least 5% total body weight loss which is statistically superior to diet and exercise control [22]. However, these criteria are yet to be demonstrated in literature with only one study partially fulfilling these criteria [19]. Given these findings, the effectiveness of BTA for weight loss remains to be elucidated clinically.

## 3 Public health response

Clearly, there is need for public education regarding the possible side-effects of getting intragastric BTA injections and for stricter and more vigilant monitoring of such procedures in medical centres. For its part, the Turkish Guidelines on Clinical Protocols for Obesity and Metabolic Surgery are clear that there is controversial and inconsistent evidence in the literature regarding the use of BTA injections for weight loss procedures. The guidelines also state the procedure has not been approved yet.^3^ Additionally, The Turkish Association of Bariatric and Metabolic Surgery also state that the patient should be notified about the current literature and efficacy of this procedure. Strongly, the association stresses that intragastric BTA injections should be administered only as part of clinical trials or scientific investigations that have been cleared by relevant research ethics committees. Deviations from these guidelines could open the medical professionals to malpractice suits.^4^

Nevertheless, the Turkish Ministry of Health has noted that the use of botulinum toxin as an approach to bariatric surgery is still practiced in many private practices throughout the country. As a response to the issue, the Turkish Association of Clinical Microbiology and Infectious Diseases has underlined the importance of increasing the stocks of botulinum antitoxin in Turkish hospitals to reserve the national capacity in approaching and treating patients suffering from botulism poisoning [23]. The British authorities have also advised their citizens to generally avoid undergoing such procedures. In cases where patients do wish to proceed with such procedures, it has been recommended that patients should be cautious and ensure that the clinic is reputable and that the products being used have CE mark (C∈) and approval for use in the home countries [24]. The Robert Koch Institute (RKI) in Germany, on the other hand, has reminded the clinicians of the obligation to report all cases of botulism to relevant authorities, even in cases of suspicion [25].

## 4 Conclusions

Though a rare event, iatrogenic botulism is a serious condition that requires constant patient monitoring and rapid administration of antitoxins. There is a need for stricter enforcement of guidelines that prohibit the off-label use of botulinum toxin for weight loss procedures. Furthermore, there is need for public education against opting for such procedures. Stricter controls and oversight of international medical tourism are needed for this and other potentially dangerous procedures.

## Ethical approval

Not applicable. All data presented in the paper has been collected from open-source platforms with proper citation and/or from media sources.

## Funding

The present paper didn't receive any external funding and was self-supported by the authors.

## Author contributions

NJ and EL conceptualized the paper whilst all authors were involved in data collection, writing, correction, and finalizing of the final draft. Visualization was done by NJ. All authors have read and agreed to the final version for publication.

## Declaration of competing interest

The authors declare that they have no known competing financial interests or personal relationships that could have appeared to influence the work reported in this paper.

## Footnotes

2 Search technique - [Botulinum toxin A] AND [obesity] on PubMed yielded 62 results on 05th May 2023. There were 8 articles (13%) that were found to have Turkish authors as corresponding authors. Furthermore, 22 articles (35%) had Turkish authors (both corresponding and non-corresponding positions). Data analysed using Bibliometrix online interface for R studio.

3 The guidelines are available from the following link [Turkish language; published 2021; online] https://shgm.saglik.gov.tr/Eklenti/41294/0/obeziteklinikprotokolu13082021pdf.pdf (accessed 05th May 2023).

4 The statement of the association is available from the following link [Turkish language; published 2019; online] http://tsmbs.org/upload/BMCD-BOTOXRapor.pdf (accessed 05th May 2023).
